# Sarcoidosis. Una manifestación infrecuente. A propósito de dos casos

**DOI:** 10.1016/j.aprim.2024.103141

**Published:** 2024-11-12

**Authors:** Esperanza Salcedo Lobera, Jessica Martínez Molina, Francisco Espildora Hernández

**Affiliations:** Servicio de Neumología, Hospital Regional Universitario de Málaga, Málaga, España

Se presenta el caso de una mujer de 41 años, fumadora con un índice acumulado tabáquico (IPA) de 15 paquetes/año sin antecedentes de interés. Acude a consulta por clínica de infección respiratoria de meses de evolución sin mejoría con tratamientos pautados. Se solicita una tomografía computarizada de tórax (TC de tórax) donde se observan adenopatías mediastínicas bilaterales con un nódulo pulmonar en lóbulo superior derecho a descartar neoplasia pulmonar (fig. 1 A), posteriormente se realiza una tomografía con emisión de positrones (PET) con aumento de captación en adenopatías mediastínicas (*Standardized Uptake Value 20*), retroperitoneales y lesiones óseas múltiples localizadas en múltiples huesos con afectación intramedular compatible con infiltración neoplásica avanzada (fig. 1 B y C). Ante dichos hallazgos, se decide realizar una ecobroncoscopia para la toma de muestras con resultado negativo para malignidad y encontrando fragmentos de granulomas no necrotizantes compatibles con sarcoidosis.

**Figura 1:**
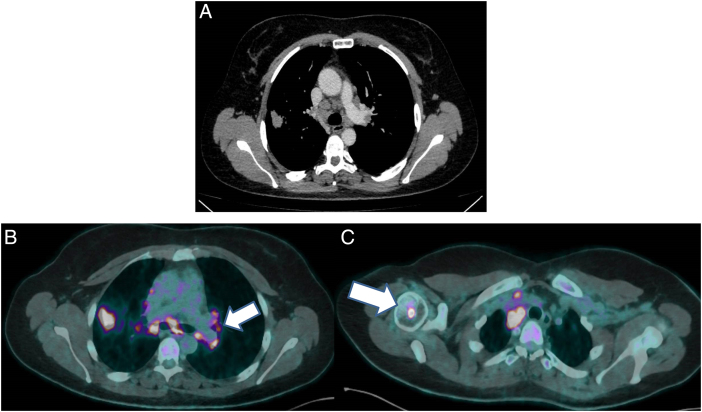
A) Corte axial en TC de tórax donde se observa una adenopatía a nivel mediastínico junto con nódulo pulmonar a nivel del lóbulo superior derecho. B) Corte axial de PET-TC con aumento de captación a nivel de adenopatías mediastínicas (flecha) junto con nódulo pulmonar. C) Corte axial de PET-TC con aumento de captación a nivel óseo (flecha). TC: tomografía computarizada; PET: tomografía con emisión de positrones.

También se presenta el caso de un varón de 44 años, fumador con un IPA de 30 paquetes /año, con trastorno esquizoafectivo en tratamiento por Salud Mental. Manifiesta una clínica de dos meses de evolución de disfagia, se realizan diferentes pruebas, entre ellas, TC de tórax con adenopatías mediastínicas bilaterales y nódulo pulmonar a nivel de lóbulo inferior derecho, en la PET se observa un aumento de captación de dichas adenopatías junto con afectación intramedular a nivel de C1, compatible con enfermedad neoplásica pulmonar avanzada, se efectúa una mediastinoscopia observándose granulomas no necrotizantes compatibles con sarcoidosis.

La sarcoidosis es una enfermedad inflamatoria que afecta a diferentes órganos, se caracteriza por la presencia de granulomas no necrotizantes en los tejidos. La localización pulmonar, tanto ganglionar como a nivel del parénquima, es la más frecuente siendo la afectación ósea una manifestación rara, de hecho, en muchas ocasiones ante estos hallazgos se sospecha en proceso neoplásico como primera aproximación diagnóstica^1^.

La peculiaridad de estos dos casos es que no presentaban clínica a nivel óseo y ante los hallazgos de las pruebas de imagen se sospechó una patología neoplásica, mientras que, en los pocos casos publicados en la literatura, hasta el 50% presentan sintomatología a nivel óseo, además es mucho más infrecuente este tipo de manifestación en ausencia de afectación cutánea^2^.

La prevalencia de este tipo de manifestación es variable en la literatura, situada en torno al 0,5%-1,5%^3^, por ello, es importante recordar este tipo de presentación de la sarcoidosis para poder realizar un diagnóstico preciso.

## Financiación

Este trabajo no ha recibido ningún tipo de financiación.

## Conflicto de intereses

Los autores declaran no tener ningún conflicto de intereses.

## References

[1] Baughman R.P., Teristein A.S., Judson M.A., Rossman M.D., Yeager H., Bresnitz E.A. (2001). Clinical characteristics of patients in a case control study of sarcoidosis. Am J Respir Crit Care Med..

[2] Sparks J.A., McSparron J.I., Shah N., Aliabadi P., Paulson V., Fanta C.H. (2014). Osseous sarcoidosis: clinical characteristics, treatment, and outcomes--experience from a large, academic hospital. Semin Arthritis Rheum..

[3] Jaber J.F., Allan R.W., Mastin S., Lowther G. (2023). Osseous Metastasis: An Unusual Sarcoidosis Masquerade. Mayo Clin Proc..

